# Preparation and immunological properties of a nanovaccine against *Pseudomonas aeruginosa* based on gold nanoparticles and detoxified lipopolysaccharide

**DOI:** 10.22038/ijbms.2020.50732.11550

**Published:** 2021-02

**Authors:** Faezeh Najafzadeh, Asghar Tanomand, Azam Haddadi, Jafar Majidi

**Affiliations:** 1 Department of Microbiology, Karaj Branch, Islamic Azad University, Karaj, Iran; 2 Department of Microbiology, Maragheh University of Medical Sciences, Maragheh, Iran; 3 Department of Immunology and Immunology Research Center, Faculty of Medicine, Tabriz University of Medical Sciences, Tabriz, Iran

**Keywords:** Gold nanoparticles, Immune response, Lipopolysaccharide, Nanovaccine, Pseudomonas aeruginosa

## Objective(s):

*Pseudomonas aeruginosa* is one of the most important bacterial pathogens in immunocompromised patients, and the lipopolysaccharide (LPS) of this organism is a key factor in virulence and both innate and acquired host responses to infection. In this study, we prepared a nanoconjugate vaccine composed of *P. aeruginosa* detoxified lipopolysaccharide (D-LPS) and gold nanoparticles (Au NPs) and evaluated its potential as a vaccine candidate against *P. aeruginosa* infections. 

## Materials and Methods:

LPS from *P. aeruginosa *strain PAO1 was extracted by the hot phenol method with some modifications and then detoxified. Au NPs were synthesized by the reduction of hydrochloroauric acid trihydrate by sodium borohydride and then coupled to D-LPS via electrostatic interaction. Mice were subcutaneously injected in the tail base with 20 µg of D-LPS, D-LPS-Au NPs, Au NPs, and PBS. IgG titers were evaluated by ELISA and whole-cell ELISA methods. The immunized and control group mice were challenged with a 2×LD_50_ (7.5×10^7^ CFU) of *P. aeruginosa* strain PAO1. 

## Results:

Mice vaccinated with D-LPS and D-LPS-Au NPs elicited a significant amount of IgG antibodies. Nanoconjugated LPS generated a significantly higher antibody titer compared with D-LPS alone. Also, immunization of mice with D-LPS-Au NPs increased survival times against challenge with 7.5×10^7^ CFU (2×LD_50_) of *P. aeruginosa *strain PAO1. 

## Conclusion:

Our results showed that the suggested vaccine composed of *P. aeruginosa* D-LPS and Au NPs had a significant potential to protect against *P. aeruginosa *infections. 

## Introduction

*Pseudomonas aeruginosa* is a gram-negative opportunistic pathogen that poses a major threat to immunocompromised individuals such as patients with cancer, burn wounds, or cystic fibrosis (1). The success of *P. aeruginosa* as a pathogen is due to its intrinsic and acquired antibiotic resistance mechanisms, infection in individuals with underlying diseases, ability to establish robust biofilms, and the production of numerous toxic products, all contribute to the difficulty in treating such life-threatening infections (2, 3). As mentioned, this bacterium has the ability to easily acquire antimicrobial resistance. The emergence of multiple drug-resistant strains has become a major clinical problem due to limited treatment options (4). Therefore, immunoprophylaxis and immunotherapy may be effective methods for controlling *P. aeruginosa* infections and due to this, development of a vaccine has become a necessity (5). A primary approach to developing a vaccine against this organism has involved searching for *P. aeruginosa* virulence factors that generate protective antibodies (6). 

An important virulence factor of *P. aeruginosa* is the LPS located in the outer portion of the outer membrane which promotes infection by interference with the host immune response (1, 7). LPS has been implicated as a critical virulence factor or protective antigen or both when examined in experimental animal models. Anti-LPS antibody has been shown to play a key role in protection against *P. aeruginosa *infections (8). Therefore, LPS is capable of acting as a target for effective immunity against *P. aeruginosa *infections (9). 

Recently, Au NPs have received attention due to their potential use over traditional vaccine platforms. The location of Au NPs in lymphoid tissues and cells, their capacity of coupling to a variety of biomolecules, their stability, ease of their synthesis, their unique physical properties (size and shape-dependent), and their safety (essential for the development and synthesis of vaccines) provide a multifaceted focus for the design of this new vaccine platform (10, 11). Furthermore, some *in vitro* and *in vivo* studies have revealed that various immune cells, including macrophages, dendritic cells, and lymphocytes, are stimulated by Au NPs leading to the production of proinflammatory cytokines (interleukin 1 β and tumor necrosis factor α) and T helper 1 cytokines (interferon γ and interleukin 2). Thus, the Au NPs can serve as both an antigen carrier and an adjuvant in immunization (12, 13). 

In this report, we describe the preparation and assessment of immunological properties of detoxified lipopolysaccharide-gold nanoparticles (D-LPS-Au NPs) conjugate, in order to determine whether conjugation onto Au NPs will enhance immunogenicity in mice. 

## Materials and Methods

***Bacterial strain and culture conditions***

*P. aeruginosa* strain PAO1 was provided by Biological Research Center, Karaj Branch, Islamic Azad University, Karaj, Iran. It was cultured on Mueller-Hinton agar medium (Merk, Germany) for 24 hr at 37 °C (14). 

***LPS purification***

The hot phenol method with some modifications was used to isolate LPS (15). After the growth of bacteria, *P. aeruginosa* colonies were cultured on nutrient broth medium (Merk, Germany) in a shaker incubator for 72 hr at 37 °C. Cells were suspended in distilled water and heated for 20 min at 66 °C, then 90% phenol (Sigma-Aldrich, China) was added and stirred for 30 min at 66 °C. After samples were rapidly cooled on ice, the suspension was centrifuged at 4000 × g for 45 min at 4 °C. The aqueous phase was removed and cold 95% ethanol was added and stored at 4 °C overnight. After centrifugation, trichloroacetic acid (TCA) (SigmaAldrich, China) (10% final concentration) was added and stirred for 30 min at 4 °C. The samples were then centrifuged and the supernatant was dialyzed against distilled water for 24 hr at 4 °C with three changes of water. The LPS was precipitated with three volumes of cold 100% ethanol and stored at 4 °C overnight. After centrifugation and collection of the pellet, purified LPS was lyophilized and stored at 4 °C (14-16). 

***LPS characterization***

The purified LPS was evaluated by sodium dodecyl sulfate-polyacrylamide gel electrophoresis (SDS-PAGE) with Coomassie blue staining. Analytical SDS-PAGE was carried out based on the method of Laemmli. Extracted LPS was dissolved in Laemmli sample buffer (Bio-Rad Laboratories, Inc., Hercules, CA, USA) and incubated at 95 °C for 5 min. Then, the sample was loaded in 4%-12% polyacrylamide gel (Mini-PROTEANVR TGX TM precast gels, Bio-Rad Laboratories, Inc.). Gel electrophoresis was performed at 60 mV for 3.5 hr (until the bromophenol blue reached the bottom of the gel). The gels were stained with the Coomassie brilliant blue R-250 staining solution (Bio-Rad Laboratories, Inc.) for 2 hr and destained overnight with the Coomassie brilliant blue R-250 destaining solution (Bio-Rad Laboratories, Inc.) (17). The protein content in the purified LPS was determined using the Bradford assay. To perform a Bradford assay, the purified LPS was added to microcentrifuge tubes and brought to a volume of 800 μl with water. Next, 200 μl of 5X Bradford reagent (Bio-Rad laboratories catalog number 500-0006) was added to the sample to bring it to a volume of 1 ml. The sample was then analyzed in a Beckman spectrophotometer to determine their absorbance at 595 nm. A standard curve was prepared with bovine serum albumin (BSA) as a control for all experiments. To do this, 2.5, 5, 7.5, 10, 12.5, 15, 17.5, and 20 μl of BSA at a concentration of 1 mg/ml was added to 800 μl of water and then used in the assay (18). LPS was analyzed for nucleic acid by measuring A_260_. The UV spectrophotometer, Nanodrop-ND1000 (Thermo Fisher Scientific, Waltham, MA), was used to measure DNA concentration, wherein 1 optical density at 260 nm is equivalent to 50 ng/μl of dsDNA (19). 

***LPS detoxification***

For LPS detoxification, the LPS pellet was dissolved in NaOH to a final concentration of 0.2 N and heated for 2 hr at 100 °C. After cooling on ice, the pH was adjusted to 7 with 1 M HCl. LPS was dialyzed extensively against distilled water for 2 days at 4 °C with six changes of distilled water. Detoxified LPS (D-LPS) was harvested by cold absolute ethanol precipitation and pooled after centrifugation at 4000 × g for 45 min at 4 °C (14, 16). 

***Pyrogenic test of D-LPS***

The toxicity of D-LPS was assayed by the Limulus amebocyte lysate (LAL) test (Lonza, Switzerland) and expressed in endotoxin units related to the U.S. standard. For pyrogenicity studies, New Zealand White rabbits (2 to 2.5 kg, 3 in each group) were administered graded doses of D-LPS intravenously. Rectal temperatures were measured with indwelling rectal thermostats and recorded every 15 min for 1 hr before the challenge and every 15 min for 3 hr after the challenge. A negative response was less than a 0.5 °C rise in temperature at any time point (20). 

***Au NPs preparation and characterization***

Hydrochloroauric acid trihydrate (HAuCl_4_ 3H_2_O) was purchased from Sigma-Aldrich Shanghai Trading Co Ltd, Shanghai, China. Au NPs were prepared with a phase-transferred process by the self-reduction method. Briefly, to a solution of HAuCl_4_ in deionized water was added trisodium citrate (Sigma, USA) under vigorous stirring. HAuCl_4_ was phase-transferred into the organic phase using sodium borohydride (NaBH_4_) (Sigma, USA). Dropwise addition of a freshly prepared aqueous solution of reduction agent (NaBH_4_) caused an instant color change. The resulting mixture was stirred at room temperature for an additional 5 hr. A wine-red organic phase containing Au NPs and a colorless aqueous phase were obtained. Therefore, the formation of gold nanoparticles was indicated by a visual color change from light yellow to wine red (21, 22). 

The particle size distribution of Au NPs was analyzed using transmission electron microscopy (TEM) micrographs (Philips, em208s, USA) at an accelerating voltage of 100 kV (17, 23). 

Dynamic light scattering (DLS) was used to determine the mean size and size distribution of Au NPs. Measurements were made with a Malvern ZEN3600 (Malvern, ZEN3600, England). Zeta potential (Z-potential) (surface charge) measurements were used to determine the stability of a colloidal suspension of electrostatically stabilized Au NPs. Conversely, DLS allows the determination of the hydrodynamic diameters of colloidal particles and conjugates, i.e., the diameter of the sphere with the same Brownian motion as the particle under analysis (17, 24). 

***D-LPS-Au NPs conjugate preparation***

Hydrogen chloride-Potassium chloride (HCl-KCl) buffer (0.02 M, pH 2) was added to the Au surface. Then D-LPS was added to the above mixture under vigorous stirring and the resulting mixture was stirred for an additional 3 hr. The surface was washed with HCL-KCl buffer. The negatively charged backbones of D-LPS adsorbed on the positively charged Au NPs through electrostatic interaction and could potentially lead to the aggregation of (+) Au NPs (25, 26). 

***Evaluation of nanoconjugate***

*Chemical analysis of nanoconjugate*

The nanoconjugate was chemically analyzed for protein level by the Bradford method and carbohydrate level by the phenol-sulfuric acid method (8, 27). To perform the phenol-sulfuric acid method, to standard carbohydrate solutions (glucose at concentrations of 10-100 μg/ml) and sample, 200 μl of 5% phenol reagent and then 1 ml of concentrated sulfuric acid were added. After 10 min, the tubes were shaken vigorously for 30 min. Then, the absorbance of the tubes at 490 nm was read and the total carbohydrate concentration in the unknown sample was obtained using a standard curve (28). 

*Pyrogenic test of nanoconjugate*

Rabbit pyrogen test for D-LPS-Au NPs was performed as previously described for D-LPS (20). The toxicity of the prepared nanoconjugate vaccine was evaluated in 5 groups of 6–8 week old female BALB/c mice (5 mice per group). Various doses of D-LPS-Au NPs (100, 150, 200, 250, and 300 μg per mouse) were subcutaneously injected in the tail base of mice.

Mice were monitored for any sign of illness or weight-loss for 7 days after the challenge (29).

*Physicochemical characterization of nanoconjugate*

The average diameter of Au NPs after conjugation onto D-LPS was determined from the TEM micrographs using a Philips em208s electron microscope at an accelerating voltage of 100 kV (17, 30). 

DLS analysis (Malvern, ZEN3600, England) was used to determine the mean size and size distribution and check on the Z-potential of Au NPs after conjugation onto D-LPS (17, 24).

Energy-dispersive X-ray spectroscopy (EDS) was used to analyze the elemental compositions of synthesized particles (31). EDS measurement was performed on a Compact Detector Unit (SAMX Detector, MIRA III, France) incorporated into a FESEM (TESCAN, Czech Republic). The EDS spectrum was obtained at an acceleration voltage of 20 kV. Mapping was completed using pseudo-color to represent the spatial distribution of energy emission from the Au element present in the sample (32). 

The characterization of the chemical groups present in NPs obtained by Fourier transform infrared (FTIR). FTIR spectra over the wavelength range of 4000–400 cm^-1^ were recorded for D-LPS and D-LPS-Au NPs using an FTIR spectrometer (30, 33). Samples were mixed with propidium iodide (PI)/water solution for a weight ratio of sample/dye of 2.6. The solution was allowed to dry to a purple powder. Samples were also dried to a thin gold film. PI powder was used as received. FTIR was performed on a Thermo AVATAR FTIR “Smart Orbit” (Thermo, AVATAR, USA). A background scan was performed prior to sample scanning. All scans were performed with 1024 scans at 4 cm^-1^ resolution (34). 

***Immunization of mice***

The 6-8 weeks old female BALB/c mice were obtained from the Laboratory Animal 

Production Department at Pasteur Institute of Iran (Research & Production Complex, Alborz). Animals care, feeding, and all experiments were performed based on institutional ethical guidelines and international protocols (35).

Mice were divided into 4 experimental groups (each group included 7 mice) and subcutaneously injected in the tail base with 20 µg of D-LPS, D-LPS-Au NPs, Au NPs (test groups), and 300 μl PBS (control group) on days 0 (with complete Freund’s adjuvant (Sigma, USA)), 14, 28, and 42 (with incomplete Freund’s adjuvant (Sigma, USA)). One week after the last injection, the mice were bled from the orbital sinus, and sera were collected and stored at – 20 °C until analysis (36).

***Enzyme-linked immunosorbent assay (ELISA)***

LPS-specific total IgG levels in sera of different mouse groups were determined by enzyme-linked immunosorbent assay (ELISA). Briefly, D-LPS, D-LPS-Au NPs, and Au NPs were diluted to 5 μg/ml with 0.1 mol/l carbonate/bicarbonate buffer (pH 9.6) and divided into 96-well flat-bottom microtiter plates (Nunc MaxiSorp, Sigma, USA) using 100 μl per well. 

After 45 min incubation at 37 °C, plates were washed twice with PBS containing 0.05% Tween 20 (PBS-T). 250 μl of blocking buffer (2% BSA in PBS-T) was added to each well to block non-specific binding sites and plates were incubated for 1 hr at 37 °C. Then, the plates were washed twice with PBS-T. 100 µl of diluted mouse sera (1:100 dilution) was added to each well and incubated for 45 min at 37 °C. After washing the plates, 100 µl of diluted horseradish peroxidase-conjugated goat anti-mouse IgG (Abcam, USA) (1:5000 dilution) was added to each well and then plates were incubated for 45 min at 37 °C. After incubation and wash steps, 100 µl of 3, 3′, 5, 5′-tetramethylbenzidine (Sigma, USA) was added to the wells. After 15 min incubation in the dark, reactions were stopped using 1 mol/L H_2_SO_4_ and the optical density (OD) was measured using an automated ELISA reader at 450 nm. ELISA test was performed in triplicate for all samples (37, 38). 

***Whole-cell ELISA***

A single colony of *P. aeruginosa* strain PAO1 was inoculated into LB broth medium and grown overnight at 37 °C with vigorous shaking. After incubation, all bacterial cells were collected, washed twice with PBS, and resuspended in PBS at a final concentration of 10^8^ CFU/ml. The bacterial suspension (100 µl/well) was coated into ELISA microtiter plates, incubated for 1 hr at 37 °C, and washed twice with PBS-T. The blocking and addition of other reagents were performed as mentioned above for ELISA (39). 

***Opsonophagocytosis assay***

The ability of vaccinated antisera to promote the uptake of *P. aeruginosa* by murine peritoneal macrophages was determined by the visual phagocytosis assay. For preparation of peritoneal macrophages, mice were injected intraperitoneally with 0.5 ml sodium thioglycollate. After 4 days, peritoneal exudate cells were harvested by washing the peritoneal cavity with 10 ml cold RPMI-1640 medium and aspirating the exudate with a syringe. The cells were washed twice with cold PBS and suspended in RPMI-1640 medium with 10% fetal calf serum (FCS), and finally, cell viability was evaluated by the dye exclusion method (40).

A mucoid colony of* P. aeruginosa* strain PAO1 was grown to mid-log phase on LB broth medium at 37 °C with shaking, washed twice with cold PBS, and suspended in PBS at a concentration of 10^6^ CFU/ml. Opsonization was performed by tumbling 100 μl of bacteria (10^6^ CFU/ml) for 30 min at 37 °C in 100 μl of heat-inactivated sera obtained from various vaccinated and control groups. After washing twice with PBS, 100 μl of 10^6^ macrophages/ml and 10% fresh infant rabbit serum as a complement source were added to the mixture and incubated for 90 min at 37 °C. The control samples consisted of tubes from which macrophage, complement, or serum was omitted. After 90 min of incubation at 37 °C, 10 μl samples were removed and diluted in PBS. Finally, the samples were plated and incubated for 18 hr at 37 °C for bacterial enumeration. The opsonophagocytosis activity of the sera was determined as follows (27, 29): 

Opsonic activity= [1-(CFU of immune serum at 90 min/ CFU of preimmune serum at 90 min)]×100 

***Challenge study***

The 50% lethal dose (LD_50_) of *P. aeruginosa *strain PAO1 was determined in 5 female BALB/c mice groups (7 mice per group, 6–8 weeks old) in order to estimate the infecting inoculum for *in vivo* protection test. Mice were intraperitoneally injected with 2.5×10^7^, 5×10^7^, 7.5×10^7^, 1×10^8^, and 12.5×10^8^ CFU of *P. aeruginosa *strain PAO1 suspended in sterile PBS. Mice were observed for 10 days, mortality was recorded, and then LD_50_ was determined according to the Reed and Muench method. For the bacterial challenge, 14 days after the last vaccination, the test and control group mice were intraperitoneally challenged with 7.5×10^7^ CFU (2×LD_50_) of *P. aeruginosa* strain PAO1 in sterile PBS. The survival rate was daily recorded in the challenged mice for 10 days. They were observed every 6 hr for the first 2 days and twice a day thereafter and mortality was recorded (29, 41). 

***Statistical analysis***

SPSS version 22.0 was used for data analysis. Differences in the mean ELISA absorbance of each group with the mean of the control group were compared by Student’s t test. Values of *P*≤0.05 were considered to be significant. The ratio of live mice/total mice (survival percent) was used for the survival study (27, 29). 

## Results

***Evaluation of D-LPS***

**Figure 1:**
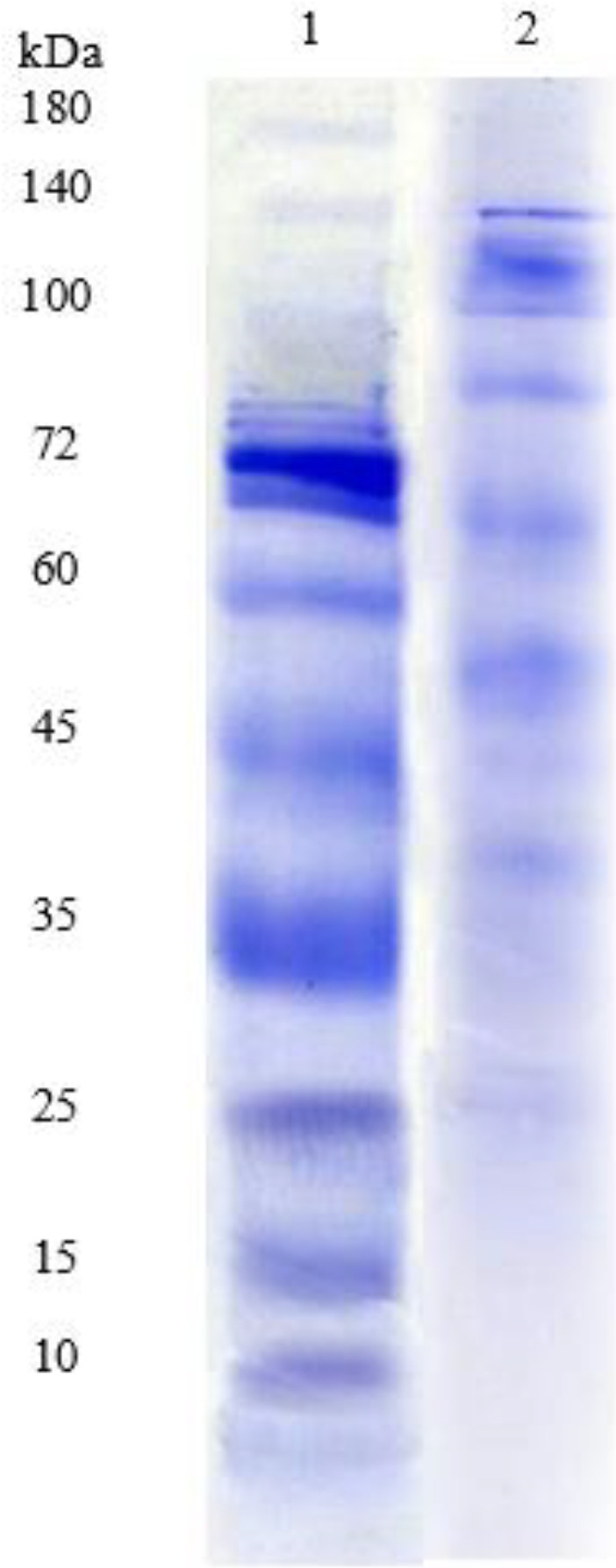
Coomassie blue-stained sodium dodecyl sulfate-polyacrylamide gel electrophoresis (SDS-PAGE) of extracted lipopolysaccharide (LPS) from *Pseudomonas aeruginosa* PAO1. Lane: 1, protein marker; 2, extracted LPS. SDS-PAGE of the purified LPS shows a progressive ladder-like pattern of bands up the gel

Coomassie blue staining of LPS from *P. aeruginosa* strain PAO1 by SDS-PAGE revealed a progressive ladder-like pattern of bands up the gel (Figure 1). For members of the family *Enterobacteriaceae* and *P. aeruginosa* strains, these bands have been reported to represent LPS molecules containing increasing lengths of O antigen (42). The protein and nucleic acid amounts of the purified LPS were 0.5 μg/ml, and 0.5 μg/ml, respectively. The amount of endotoxin in LPS, estimated by the LAL test, was 0.125 endotoxin units per ml. There were not pyrogenic in rabbit thermal tests. 

***Evaluation of nanoconjugate***

The protein and carbohydrate contents of D-LPS-Au NPs were 0.5 μg/ml, and 0.4 μg/ml, respectively. D-LPS-Au NPs conjugate was non-pyrogenic and evoked <0.5 °C increase in body temperature of each rabbit after 24 hr. Toxicity test of D-LPS-Au NPs showed that none of the mice injected with various doses of nanoconjugate (between 100 and 300 μg) died after 7 days. 

**Figure 2:**
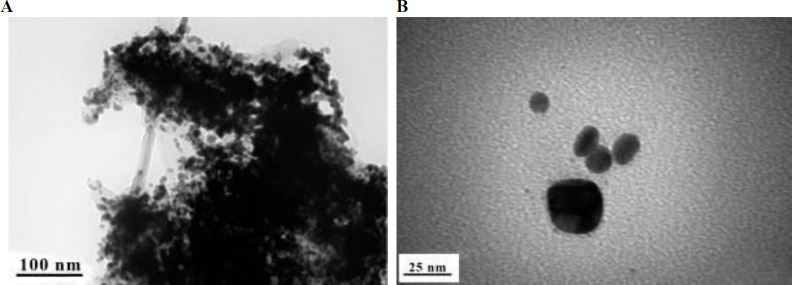
Transmission electron microscopy (TEM) images of gold nanoparticles (Au NPs) before (A) and after conjugation onto detoxified lipopolysaccharide (D-LPS) (B). TEM micrographs illustrating the size and morphology of Au NPs before and after conjugation onto D-LPS. The average size of Au NPs after conjugation onto D-LPS is 14 nm (B). The scale bar (A) 100 nm, (B) 25 nm

Au NPs before and after conjugation onto D-LPS were imaged using TEM (Figure 2). Au NPs after conjugation onto D-LPS had a mean diameter of 14 nm (Figure 2B). 

**Figure 3:**
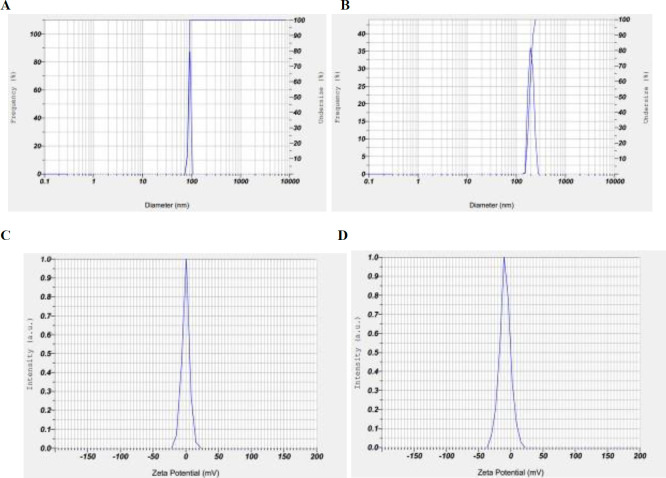
Characterization of gold nanoparticles (Au NPs) before and after conjugation onto detoxified lipopolysaccharide (D-LPS). The size distribution of the intensity obtained from dynamic light scattering (DLS) measurements of Au NPs (A) and D-LPS-Au NPs (B). Zeta potential (Z-potential) for Au NPs (C) and DLPS-Au NPs (D). The increase in Au NPs diameter (A and B) and the decrease in Z-potential (C and D) indicated binding of D-LPS to the Au NPs

D-LPS binding to Au NPs was assessed by DLS and Z-potential in comparison with Au NPs (Figure 3). As shown in Figures 3A and B, Au NPs showed a size increase via electrostatic interaction to D-LPS (Figures 3A and B). In addition to increased Au NPs diameter after conjugation, a decrease in Z-potential indicates D-LPS binding to the Au NPs (Figures 3C and D). 

**Figure 4:**
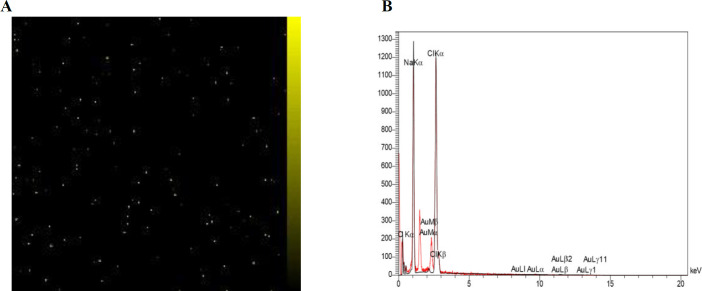
Energy-dispersive X-ray spectroscopy (EDS) mapping (A) and spectrum (B) of detoxified lipopolysaccharide (D-LPS)-gold nanoparticles (Au NPs). EDS mapping (A) demonstrates that Au NPs (the yellow dots represent Au) were loaded as a homogeneous dispersion in the sample. In the EDS spectrum (B), peaks are labeled and Au is detected

EDS mapping analysis demonstrates that Au NPs were loaded as a homogeneous dispersion in D-LPSAu- NPs (Figure 4A). The EDS spectrum of D-LPS-Au NPs showed Au signals, which confirmed the presence of elemental Au in the sample (Figure 4B). 

**Figure 5:**
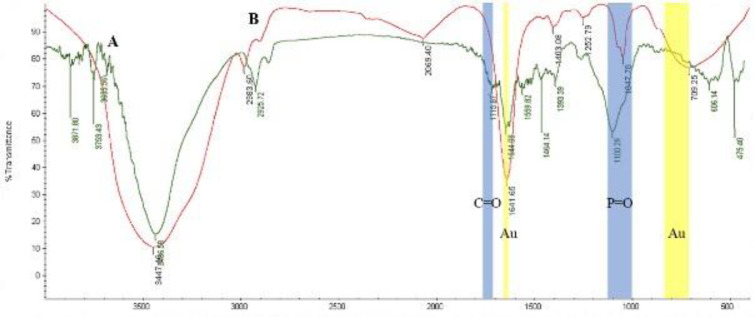
Fourier transform infrared (FTIR) spectra recorded from detoxified lipopolysaccharide (D-LPS) (spectrum A) and D-LPS-gold nanoparticles (Au NPs) (spectrum B) in the range 4000–400 cm^−1^. Blue areas 1120–1000 cm^-1^ and 1760-1710 cm^-1^ of spectrum B are related to the formation of P=O and C=O bonds, respectively, and these spectra confirmed the binding of Au NPs to D-LPS. The expansion of spectrum B in yellow regions 840-710 cm^-1^ and 1641 cm^-1^ are related to Au NPs

FTIR spectroscopy was used for structural analysis of the conjugated NPs and conjugation bonds. Figure 5 shows FTIR spectra of D-LPS (spectrum A) and D-LPS- Au NPs (spectrum B) in the spectral region 4000–400 cm^−1^. As seen in Figure 5, peaks spreading in the areas of 1120–1000 cm^-1^ and 1760-1710 cm^-1^ are related to the formation of P=O and C=O bonds, respectively, and these spectra confirmed the binding of Au NPs to D-LPS. The expansion of the FTIR spectrum of the nanoconjugate in regions 840-710 cm^-1^ and 1641 cm^-1^ are related to the Au NPs (Figure 5). 

***ELISA of immune responses***

One week after the last injection, the levels of IgG against D-LPS, D-LPS-Au NPs, and Au NPs were measured by indirect ELISA and whole-cell ELISA methods. The antibody levels were compared with each other. Comparisons of experimental groups were carried out at 1:100 antibody dilution. 

In indirect ELISA, both D-LPS and D-LPS-Au NPs elicited a statistically significant IgG antibody response but neither Au NPs nor PBS evoked antibody (*P*≤0.05) (Table 2). As shown in Table 2, D-LPS-Au NPs displayed higher titer in the IgG antibody than D-LPS (*P*≤0.05). 

Similar results were obtained using the whole bacterial cell as a coating antigen in ELISA, where the highest titer was observed in mice immunized with D-LPS-Au NPs (*P*≤0.05) (Table 2). D-LPS also indicated a Lower antibody titer than D-LPS-Au NPs. Additionally, neither Au NPs nor PBS elicited antibody (*P*≤0.05). 

***Opsonophagocytosis assay***

**Figure 6:**
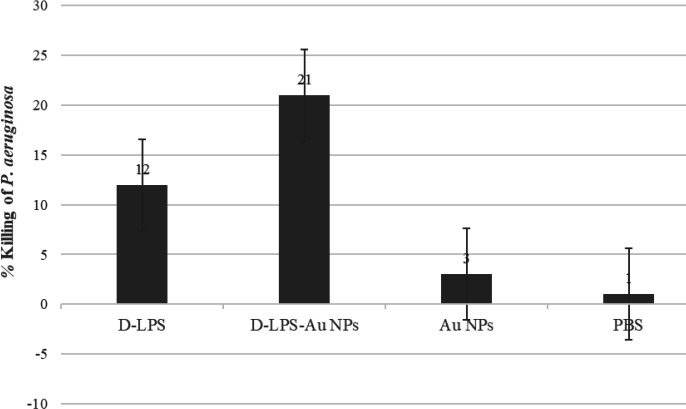
The opsonophagocytic activity of antisera from mice immunized with detoxified lipopolysaccharide (D-LPS), D-LPS- gold nanoparticles (Au NPs), Au NPs, and PBS against *Pseudomonas aeruginosa* strain PAO1. Bars represent the mean percent of Pseudomonas aeruginosa killed by sera of immunized and control group mice. mean±standard deviation

The ability of antibodies induced by D-LPS, D-LPS-Au NPs, Au NPs, and PBS to promote the uptake and killing of mucoid *P. aeruginosa *strain PAO1 is shown in Figure 6. Immunization with D-LPS-Au NPs evoked a substantial opsonic antibody response against *P. aeruginosa *strain PAO1 in comparison with other groups. There was no phagocytic killing when antibody, complement, or macrophages were omitted.

***Challenge study***

**Figure 7:**
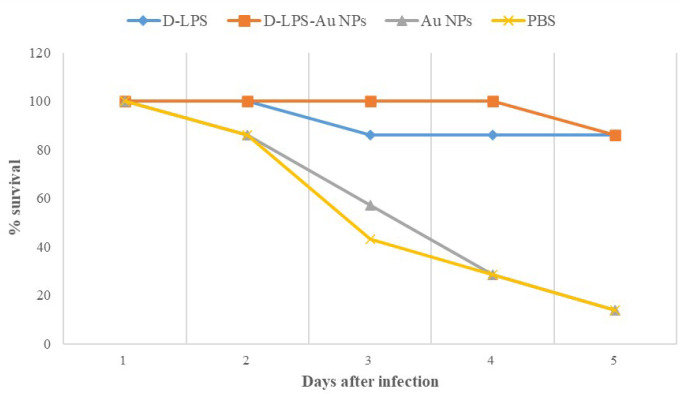
Survival rate of immunized and control group mice after challenge with 7.5×10^7^ CFU (2×LD50) of Pseudomonas aeruginosa strain PAO1. Immunizations of mice with detoxified lipopolysaccharide (D-LPS) and D-LPS-gold nanoparticles (Au NPs) show significant protection against challenge with 7.5×10^7^ CFU of Pseudomonas aeruginosa strain PAO1. Immunization with D-LPS-Au NPs also increases the survival time of mice against challenge with 7.5×10^7^ CFU of Pseudomonas aeruginosa strain PAO1

The LD_50_ for *P. aeruginosa* PAO1 was determined according to the Reed and Muench method and was found to be 3.75×10^7^ CFU and 7.5×10^7^ CFU was selected as 2 × LD_50_. Mice immunized with D-LPS and D-LPS-Au NPs showed significant protection against intraperitoneal challenge with 7.5×10^7^ CFU (2×LD_50_) of *P. aeruginosa* strain PAO1. This challenge dose killed 6/7 of mice that were injected with Au NPs and PBS, and 1/7 of mice immunized with D-LPS and D-LPS-Au NPs. Comparison of mice mortality times in immunized groups with D-LPS and D-LPS-Au NPs showed that immunization with D-LPSAu NPs increased the survival time of mice against intraperitoneal challenge with an approximate 7.5×10^7^ CFU (2×LD_50_) of *P. aeruginosa* strain PAO1 (Figure 7). These results are in good correlation with the IgG titers and also with opsonophagocytosis assay of the sera. 

**Table 1:** Size characterizations by dynamic light scattering (DLS) and Zeta potential (Z-potential) values of gold nanoparticles (Au NPs) before and after conjugation onto detoxified lipopolysaccharide (D-LPS)

Sample	Size				Z-potential	
	S. P. Area Ratio	Mean	S. D.	Mode	Z-potential	Electrophoretic mobility
Au NPs	1.00	86.2 nm	3.4 nm	86.7 nm	-0.3 mV	-0.000002 cm^2^/Vs
D-LPS-Au NPs	1.00	189.1 nm	22.0 nm	184.0 nm	-9.0 mV	-0.000070 cm^2^/Vs

**Table 2:** Levels of IgG in collected sera of BALB/c mice immunized with detoxified lipopolysaccharide (DLPS), D-LPS-gold nanoparticles (Au NPs), Au NPs, and PBS as control group at a single dilution of 1:100 in sera collected. * indicates a statistically significant difference compared with PBS as the control group, at *P*≤0.05 level

Immunogen	Target antigen	OD_450_	Mean difference	*P-value*	95% Confidence interval	
					Lower	Upper
D-LPS	D-LPS	1.646	0.37	< 0.05*	1.15	1.85
	Whole bacteria	1.86	0.48	< 0.05*	1.26	2.15
D-LPS-Au NPs	D-LPS-Au NPs	2.968	0.62	< 0.05*	2.24	3.40
	Whole bacteria	2.35	0.23	< 0.05*	1.98	2.42
Au NPs	Au NPs	0.263	0.16	> 0.05	0.17	0.41
	Whole bacteria	0.37	0.19	> 0.05	0.2	0.55
PBS	Each of the target antigens	0.145	-	-	-	-
	Whole bacteria	0.14	-	-	-	-

## Discussion

*P. aeruginosa* is an important cause of nosocomial infections leading to significant morbidity and mortality (29). Effective treatment and control of *P. aeruginosa *infections remain a persistent problem, because of the natural resistance of the bacterium and its remarkable ability to acquire resistance to multiple antimicrobial agents (43). Due to antibiotic resistance, the development of a vaccine against *P. aeruginosa* is an appropriate and challenging strategy to pursue (44). 

The LPS of *P. aeruginosa* is a key factor in virulence and both innate and acquired host responses to infection. Previous studies established that the conserved epitope of LPS in this organism had efficacy in inducing cytokine responses. In addition, LPS can bind to Toll-like receptor 4 (TLR4) on the surface of immune cells and stimulate inflammatory immune responses (9, 45). Despite extensive studies, LPS-based vaccines are not successful owing to their low immunogenicity (43). To overcome this problem, we conjugated D-LPS onto Au NPs to produce an effective nanovaccine against *P. aeruginosa* infections. For the first time, Au NPs were used as carriers of haptens for antibody preparation in 1986 (46). Since then, many studies have been focused on Au NPs as an antigen carrier and adjuvant in immunization for the development of effective vaccines (47-50). 

In this study, the LPS from *P. aeruginosa* strain PAO1 was investigated for a nanovaccine preparation, we extracted the LPS by the modified hot phenol with TCA procedure. A study showed that among extraction methods of LPS (extraction by butanol with enzymatic digestion, hot phenol extraction with enzymatic digestion, and modified hot phenol with TCA procedure), the yield of LPS extraction in the modified hot phenol with TCA procedure is 1.5 times the yields of the other two methods and accordingly this procedure is the best method for LPS production (15). 

The Au NPs were prepared by using the Natan method. To produce Au NPs, Brown *et al.* introduced a method using citrate as a stabilized agent and borohydride as a reducing agent. The Au NPs were obtained by adding the NaBH_4_/citrate mixture into the HAuCl_4_ solution at room temperature. With this method, the size of Au NPs is tailored to 6 nm (51). Then we coupled the synthesized Au NPs to D-LPS via electrostatic interaction. 

After vaccination of mice, specific IgG titers were determined by ELISA and whole-cell ELISA methods. Abu-baker *et al.* used both ELISA and whole-cell ELISA methods to measure specific IgG anti-*P. aeruginosa* LPS antibody levels in collected sera from mice immunized with O-polysaccharide recombinant exoprotein A conjugate and similar results were obtained using both ELISA methods (39). Similarly, in this study, the whole-cell ELISA was used to ensure that the LPS orientation did not change during the extraction procedure. Although we used two different coating antigens (whole bacteria and LPS), we obtained similar results with respect to the antibody titer. This indicates that the LPS isolation procedure did not change the original state of the molecules.

Our results show that D-LPS conjugated to Au NPs induced antibody responses which were superior to the responses induced by D-LPS alone. Since the coupling chemistry is selective for nucleophilic carbonyl groups in the LPS core region, this leaves the O-antigen and lipid A moieties exposed. Whilst LPS O-antigen provides epitopes for the development of antigen-specific antibodies, the lipid A region is a potent agonist for TLR4 and evokes a strong immune response from interaction with B memory cells. Additionally, the increased response from D-LPS-Au NPs may be due to the presentation of LPS in its coupled form and the high density of O-antigen epitopes localized on an Au NP surface compared with uncoupled DLPS alone (48). Other workers have reported the ability of Au NPs to enhance the ability of an antigen to evoke antibody responses compared with antigen given alone. Gregory *et al.* covalently coupled Au NPs with one of three different protein carriers TetHc (Hc fragment of tetanus toxin), Hcp1 (hemolysin co-regulated protein produced by both *Burkholderia mallei* and* Burkholderia pseudomallei*) and FliC (flagellin produced by *B. pseudomallei*)) followed by conjugation to LPS purified from a nonvirulent *B. thailandensis* strain. Mice were immunized three times intranasally. When they compared with LPS alone, the Au NPs-protein-LPS conjugates were found to generate significantly higher antibody titers (48). A similar approach was developed for vaccination against *B. pseudomallei* myeloidosis. Resaerchers immunized mice three times subcutaneously with Au NPs glycoconjugate vaccines composed of *B. thailandensis* LPS conjugated to the novel flagellar protein FlgL and a protein combination (FlgL, Hcp1, and hemagglutinin). Mice vaccinated with Au NPs glycoconjugate vaccines generated significantly higher antibody titers than did native antigens (52). Another study examined the effects of LPS adsorption on the NP surface on the formation of a biocorona in biological fluids and on the subsequent inflammation-inducing activity of NPs. Different Au NPs were exposed to *Escherichia coli* LPS under different conditions. LPS coated Au NPs, but not the LPS-free NPs, induced significant inflammatory responses in human monocytes *in vitro* (17). A study investigated the effect of intraperitoneal treatment using Au NPs on the inflammatory response and pulmonary oxidative stress in Wistar rats induced by LPS. The results showed that the LPS + Au NPs group exhibited a significant decrease in the levels of the pro-inflammatory cytokines, activities of antioxidant enzymes, oxidative stress parameters, and total leukocyte proliferation compared with the LPS group (53). 

In the opsonophagocytic killing assay, D-LPS conjugated to Au NPs was superior to D-LPS alone in its ability to induce opsonic antibodies in mice. The results found here were consistent with the findings of Safari *et al.* They reported sera obtained from mice immunized with Au glyconanoparticles, bearing a synthetic tetrasaccharide epitope related to the *Streptococcus pneumoniae* type 14 capsular polysaccharide, were able to opsonize *S. pneumonia* type 14 bacteria (54). 

Immunization with D-LPS and D-LPS-Au NPs afforded significant protection against challenge with *P. aeruginosa* strain PAO1 in comparison with Au NPs and control groups. 

However, there was no significant difference between the survival of mice immunized with D-LPS or D-LPS-Au NPs, but the survival times of mice immunized with the D-LPS-Au NPs were longer than the survival times of mice immunized with D-LPS. These findings were consistent with the results reported by Gregory *et al.* Three weeks after the final immunization boost, they tested immunized mice with Au NPs-one of three different protein carriers (TetHc, Hcp1, and FliC)-*B. thailandensis* LPS conjugates for their protective capacity against 3.5 × LD_50_ of *B. mallei* challenge and recorded the meantime to death. Gregory *et al.* indicated that there was no significant difference between the survival of immunized mice with LPS, Au NPs-LPS, or Au NPs-TetHc-LPS (48). 

## Conclusion

In general, our results showed that coupling *P. aeruginosa* D-LPS onto Au NPs markedly increased both the levels of IgG and opsonic antibodies and the survival times of immunized mice. Therefore, D-LPS-Au NPs can be used as a candidate vaccine against *P. aeruginosa* infections. 
